# *Staphylococcus aureus* Biofilm: Morphology, Genetics, Pathogenesis and Treatment Strategies

**DOI:** 10.3390/ijerph18147602

**Published:** 2021-07-16

**Authors:** Muhammad Idrees, Sheeba Sawant, Nazira Karodia, Ayesha Rahman

**Affiliations:** Faculty of Science and Engineering, University of Wolverhampton, Wolverhampton WV1 1LY, UK; M.Idrees@wlv.ac.uk (M.I.); S.Sawant2@wlv.ac.uk (S.S.); nazira.karodia@wlv.ac.uk (N.K.)

**Keywords:** *Staphylococcus aureus*, biofilm formation, gene expression, quorum sensing, antimicrobial resistance, pathogenesis, antibiofilm agents

## Abstract

*Staphylococcus aureus* is a nosocomial bacterium causing different infectious diseases, ranging from skin and soft tissue infections to more serious and life-threatening infections such as septicaemia. *S. aureus* forms a complex structure of extracellular polymeric biofilm that provides a fully secured and functional environment for the formation of microcolonies, their sustenance and recolonization of sessile cells after its dispersal. *Staphylococcus aureus* biofilm protects the cells against hostile conditions, i.e., changes in temperature, limitations or deprivation of nutrients and dehydration, and, more importantly, protects the cells against antibacterial drugs. Drugs are increasingly becoming partially or fully inactive against *S. aureus* as they are either less penetrable or totally impenetrable due to the presence of biofilms surrounding the bacterial cells. Other factors, such as evasion of innate host immune system, genome plasticity and adaptability through gene evolution and exchange of genetic material, also contribute to the ineffectiveness of antibacterial drugs. This increasing tolerance to antibiotics has contributed to the emergence and rise of antimicrobial resistance (AMR), a serious problem that has resulted in increased morbidity and mortality of human and animal populations globally, in addition to causing huge financial losses to the global economy. The purpose of this review is to highlight different aspects of *S. aureus* biofilm formation and its overall architecture, individual biofilm constituents, clinical implications and role in pathogenesis and drug resistance. The review also discusses different techniques used in the qualitative and quantitative investigation of *S. aureus* biofilm and various strategies that can be employed to inhibit and eradicate *S. aureus* biofilm.

## 1. Introduction

*Staphylococcus aureus* (also denoted as *Staph. aureus* or *S. aureus*) is a Gram-positive pathogenic bacterium and is a major cause of different infectious illnesses in humans and animals [1,2]. These illnesses may range from simple skin and soft tissue infections to more serious and life threatening conditions such as blood infections (bacteraemia/septicaemia) [3]. *Staphylococcus aureus* secretes an extracellular polymeric substance (EPS), known as biofilm, that helps the microbe to resist and minimise the effect of antibacterial drugs [4]. 

Similar to any other bacterial biofilm, a *Staphylococcus aureus* biofilm also has two distinct components, i.e., water (about 97%) and the organic matter which includes EPS and microcolonies [5]. The EPS constitutes about 50 to 90% of the total organic matter of a biofilm and is a complex of different polymeric substances, such as extracellular DNA (eDNA), proteins and polysaccharides [6,7]. The remaining portion, 10–25%, consists of microcolonies [5]. 

In *Staphylococcus aureus* biofilm, the major component of EPS is the polysaccharide intercellular adhesin (PIA) [8]. The polysaccharide component of EPS has been given the name PIA due its function, i.e., intercellular adhesion of bacterial cells, and poly-β(1-6)-N-acetylglucosamine (PNAG), due to its chemical composition. PIA are cationic in nature and play a significant role in colonisation, biofilm formation and biofilm-related infections, immune evasion, resistance to antimicrobials and phagocytosis [9].

*Staphylococcus aureus* EPS also contains a range of proteins including accumulation associated proteins (Aap), surface binding protein A (Spa), fibrinogen binding protein (FnBP) A and B, extracellular matrix binding protein (Embp), amyloid fibres and *S. aureus* surface binding protein (SasG) [10,11]. Other *S. aureus* proteins that are found covalently attached to cell wall peptidoglycan (PG) by trans peptidases (sortases) are known as cell wall-anchored proteins (CWP) [12]. There are as many as 25 different CWPs, categorised as microbial surface component recognising adhesive matrix molecule (MSCRAMM), near iron transporter (NEAT), three-helical bundle and G5-E repeat proteins [12,13]. These *S. aureus* proteins perform different functions. For example, accumulation associated protein Aap interacts with PIA and plays a role in biofilm maturation [8]. SasG protein and surface binding protein A are responsible for surface attachment and causing infections [10]. CWA proteins facilitate adhesion to EPS, to host surface, and their interaction with CWA proteins on adjacent cells contributes to the accumulation of biofilm [13].

Similarly, amyloid fibres act as a scaffold that keeps *S. aureus* cells anchored to the biofilm matrix and thus maintain the stability of the biofilm [7,14]. Alongside PIA and EPS proteins, the third important component of *S. aureus* biofilm EPS is eDNA. eDNA has been reported to be involved in irreversible attachment, horizontal gene transfer, maintaining biofilm integrity, antimicrobial resistance and host immune system evasion [15].

The extra polymeric substance of a biofilm also contains charged (both positive and negative) groups and hydrophobic groups. The negatively charged groups found in EPS include carboxyl groups, phosphates, sulphates, glutamic acid and aspartic acid, while positively charged ones include amino sugars [16]. Despite of the presence of both positively and negatively charged species, the overall charge on the EPS surface is negative and thus can serve as a better target for positively charged moieties [17,18].

## 2. Biofilm Formation

The formation of biofilm proceeds through four different stages [19], which are

1. Attachment of planktonic cells to the surface (either a biotic host or any abiotic surface);
2. Colonisation and biofilm formation;
3. Biofilm maturation;
4. Biofilm dispersal.

Biofilm formation in *S. aureus* is initiated when free floating, planktonic cells attach to the available surface and start colonising [20]. *S. aureus* adherence to a surface is influenced by hydrophobic and hydrophilic interactions between the *S. aureus* cell surface and any biotic or abiotic surface [21,22,23]. It has been found that the *S. aureus* cell surface adheres to hydrophobic surfaces by the help of many weakly binding macromolecules, while its adherence to hydrophilic surfaces involves fewer but stronger binding macromolecules [23,24]. 

**Figure 1:**
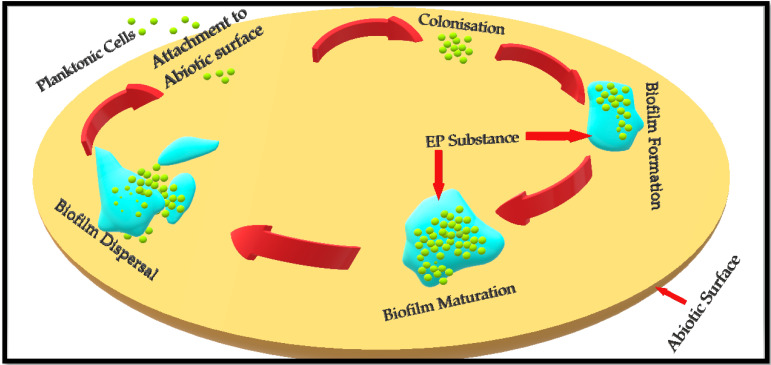
Depiction of *Staphylococcus aureus* biofilm formation on an abiotic surface. Basic concept has been adopted from Idrees et al. (2020) and Paharik (2016) [7,26].

The formation of microcolonies is followed by the formation of an extracellular polymeric substance (EPS) that develops into a fully matured biofilm [19]. Once the biofilm is fully matured, the bacterial cells residing inside it release certain chemicals, i.e., D-amino acids and EPS-degrading enzymes such as alginate lyase, to break and disperse the biofilm [25]. These planktonic cells are ready to either recolonise the same site or attach to a different site and repeat the process to form a new biofilm [6]. Figure 1 depicts different stages involved in the formation of a bacterial biofilm.

*Staphylococcus aureus* cells that are encased and protected by biofilms show different phenotypic characters compared to cells in their planktonic form. Biofilm-associated *Staphylococcus* *aureus* cells are more resistant to antibiotics and exhibit differences in cell size and growth, gene expression and protein production, compared to their free living counterparts [24]. 

Biofilm-associated *S.* *aureus* cells have been reported to have four different metabolic states, i.e., they can either be growing aerobically, can be fermentative, can be dormant, or can even be dead [27]. Besides the extracellular polymeric matrix that shelters the cells against antibiotics, the dormant and metabolically slow growing cells have also been reported to add to antimicrobial resistance [28]. Moormeier [29] reported that *S. aureus* cells encased in a biofilm grow at different rates, i.e., some cells grow at a faster rate as compared to other cells within the same biofilm. These cells are smaller in size and attain their normal size once released upon the dispersal of the biofilm. 

Biofilm associated *S. aureus* cells exhibit altered gene expressions, i.e., up- and downregulation of genes has been witnessed in the cells residing inside a *S. aureus* biofilm. The differential gene expression accounts for the variation in cell sizes within a biofilm, their growth rates and protein production [6]. 

## 3. Gene Expression during *Staph. aureus* Biofilm Formation and Dispersal

**Figure 2:**
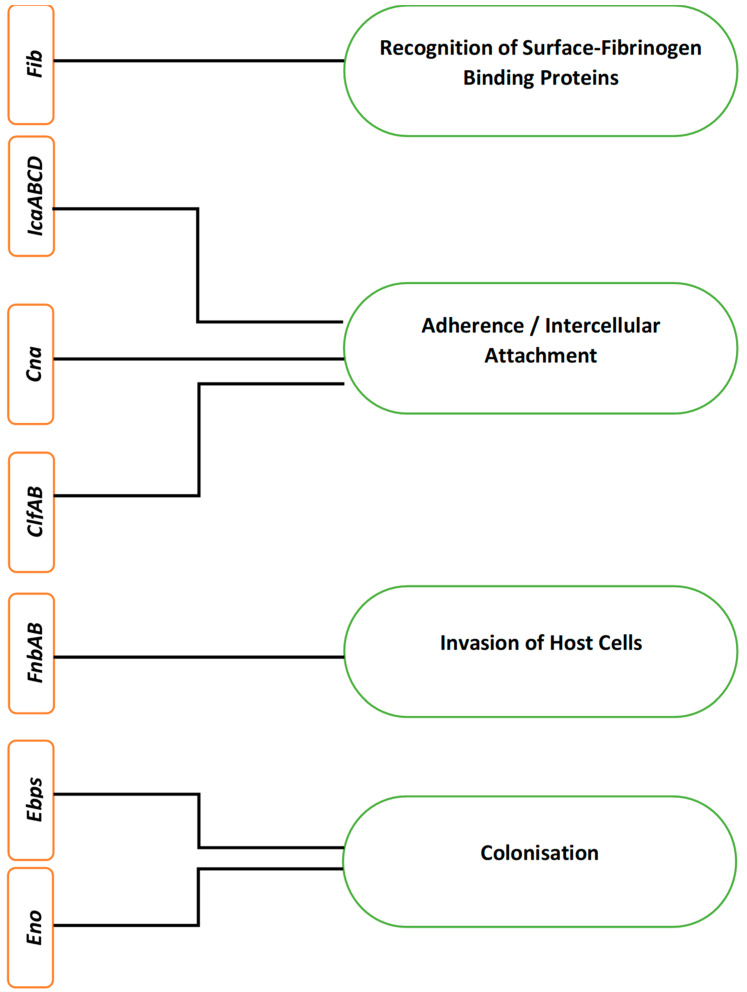
Different genes and their corresponding encoding in *Staphylococcus aureus* biofilm formation.

Like many other microbial functions, microbial biofilm formation is also encoded by certain biofilm-associated genes [30]. In *S. aureus*, biofilm formation is mainly encoded by 12 different genes, i.e., fibrinogen-binding proteins (*fib*) gene, fibronectin-binding proteins (*fnbA* and *fnbB*) genes, intercellular adhesion (*icaA, B, C* and *D*) genes, clumping factor (*clfA* and *B*), elastin binding protein (*ebps*), laminin binding protein (*eno*) and collagen binding protein (*cna*) gene [31]. Figure 2 lists different genes encoding the corresponding stages during *S. aureus* biofilm formation.

The aforementioned genes encode different surface proteins to cause *S.* *aureus* adherence to the host, its penetration into the host and its colonisation, ultimately leading to biofilm formation and virulence. In *S. aureus*, the *fib* gene facilitates and encodes the recognition of surface-fibrinogen binding proteins, while the collagen binding proteins encoded by their corresponding *cna* genes promote adherence to the surface [26]. 

The intercellular adherence genes *icaABCD* encode the process of cell to cell adherence and initiate biofilm formation [32,33]. The co-expression of *fnbAB* genes also facilitates the formation of biofilm in *S. aureus*. It has also been found that although *fnbA* and *fnbB* genes in *S. aureus* are not involved in the process of adherence, they still contribute to the development of biofilm, i.e., *fnAB* act as invasins and facilitates *S. aureus* to penetrate into the host cells [34,35].

Clumping factor genes *clfA* and *clfB* encode cell wall-anchored proteins that attach to the surface fibrinogen of the host [36]. This attachment of clumping factors A and B encoded by *clfAB* genes facilitates *S. aureus* colonisation of the host, promotes biofilm formation and causes virulence by means of immune evasion through binding soluble fibrinogen [13,37]. Serine-aspartate repeat factors C and D (SdrCD) facilitate attachment to desquamated epithelial cells and nasal colonisation, while SdrE causes immune evasion by binding to complement factor H [13]. These proteins are encoded for their respective roles by the corresponding s*dr* genes [38]. The encoding of elastin-binding proteins and laminin-binding proteins by their respective genes, *ebps* and *eno* genes, facilitates colonisation of the host and biofilm formation [39]. 

Once the biofilm is fully matured, it disperses and releases the sessile cells which are ready to repopulate either the same site (primary site) or a new site (secondary). This dispersal of the *Staphylococcus aureus* biofilm is regulated by four different genes of the accessory gene regulatory (Agr) system [29]. The *Agr* genes encoding the dispersal of biofilm include *AgrA*, *AgrB, AgrC* and *AgrD* [40]. Agr-regulated dispersal of biofilm is brought about by the induction of different proteases and phenol-soluble modulins (PSMs) [28]. These proteases and PSMs disperse the biofilm by acting as surfactants [41,42].

### 3.1. Gene Expression and Quorum Sensing in Staphylococcus aureus

Cell to cell communication in *Staphylococcus aureus* is regulated by the accessory gene regulator (Agr), a system that prompts genetic adaptations for communication in bacterial cells [40]. *Staphylococcus aureus* has a distinctive Agr system that regulates the expression of different toxins and virulence factors and controls the bacterial-host interaction at the site of infection as well [43]. 

The Agr system consists of two pairs of genes, i.e., *AgrA-AgrC* and *AgrB-AgrD*, and activates in response to the secretion of an autoinducing peptide (AIP) [40,44]. Genes *AgrB* and *AgrD* regulate the expression and transportation of AIP. Once a sufficient amount of AIP is aggregated in the surrounding extracellular environment, the two-component system of *AgrA* and *AgrD* becomes activated, triggering the intracellular communication, quorum sensing [45,46].

### 3.2. Gene Expression and Staphylococcus aureus Pathogenesis 

Pathogenesis in *Staphylococcus aureus* is a result of the expression of different virulence factors such as toxins, immunomodulators and exoenzymes [47]. The expression of these factors is regulated and encoded by particular genes [48]. Amongst the virulence factors in *S. aureus*, toxins are the most important factors that protect the bacterium by averting any possible elimination by its host’s defence system [49]. 

The expression of different toxins encoded by their corresponding genes causes different infectious morbidities. For instance, toxic shock syndrome toxin1 (TSST1), when encoded by *tstH* gene, causes toxic shock syndrome, while exfoliative toxin causes scalded skin syndrome when encoded by its corresponding *eta* and *etb* genes. Similarly, haemolysin and leukotoxin, regulated by *hla* and *lukDE* genes, are responsible for pore formation and membrane destruction in their hosts [49,50].

Exoenzymes such as lipases, proteases, nucleases, etc., encoded by genes associated with the Agr system, cause tissue destruction and metastatic infections [46,51]. The immunomodulators such as leucocidin, phenol-soluble modulins, etc., are responsible for skin invasive infections, abscesses and pneumonia when regulated by their corresponding genes, *AgrA* and *AgrC* [41]. 

Similarly, *S. aureus* pathogenesis involves evasion of the host’s innate immune system. *S. aureus*’s entry into subepidermal tissues or blood is encountered by the host immune system [52]. *S. aureus* counterattacks the host immune system and inactivates it by secreting different proteins, encoded by two immune evasion gene clusters, IEC1 and IEC2 [53].

Apart from the aforementioned virulence factors, there are other factors, such as attachment to the host and persistence factor (involved in biofilm formation), which, when regulated by their corresponding genes, cause different infectious diseases. Virulence factors involved in *S. aureus* attachment to the host surface, regulated by *clfAB*, *fnbAB*, *cna* and *ica* genes, and those involved in persistence, encoded by *ica* locus and *hemB* genes, are responsible for endocarditis, septic arthritis, cystic fibrosis and relapsing infections [47]. 

Various virulence factors, their corresponding regulatory genes and their clinical implications have been summarised in Table 1.

## 4. *Staphylococcus aureus* Biofilm and Antimicrobial Resistance (AMR)

Antimicrobial resistance (AMR) is a serious threat to human and animal lives, as many of the traditional antimicrobial drugs are losing their efficacy (partial or full). According to World Health Organisation (WHO)’s report from 2019, AMR is causing 700,000 mortalities each year, and 230,000 people die as a direct consequence of resistance to antituberculosis drugs alone [54]. Apart from being a major cause of morbidity and mortality, both in humans and animals, AMR is also causing a huge financial loss to the global economy [55].

Similar to any other biofilm-forming bacteria, *S. aureus* also develops a biofilm to slow down or prevent the diffusion of antimicrobial drugs, hence hindering drugs’ access to the cells residing inside the biofilm [56]. The formation of biofilm is prompted by different factors such as nutrient deprivation, changes in temperature (too low or too high) and dehydration [57,58]. Since biofilm provides protection to the encased cells, *S. aureus* has therefore adopted it as one of its useful tools to resist antibiotics that are generally bactericidal to the cells in their free-floating planktonic form [59]. Bacterial cells that are covered in a biofilm have been reported to resist antibiotics 10–1000 times more compared to their corresponding planktonic forms [8]. 

*S. aureus* is generally a commensal bacterium, colonising up to 30% of the human population (it colonises host skin, nostrils, armpits, and groin) globally, and many times, it causes little or no harm at all [60,61]. However, these niches of usual occurrence can sometimes become a primary cause of *S. aureus* infections [24]. Being a nosocomial pathogen, and due to its ability to form biofilms on both biotic and abiotic surfaces, *Staphylococcus aureus* infections are complicated and difficult to eradicate, particularly those caused by a methicillin-resistant strain (MRSA) [62,63]. Individuals with certain skin conditions, such as atopic dermatitis (AD), are highly susceptible to *S. aureus* colonisation and hence its biofilm formation [64,65]. Antibacterial drugs become less penetrable across the biofilm, allowing *S. aureus* cells to survive in the presence of drugs at reduced concentrations [56]. In addition to the major role of bacterial biofilms in drug resistance, other factors, such as evasion of innate host immune system, genome plasticity and adaptability through gene evolution and exchange of genetic material, also contribute to the development and rise of AMR [24,66]. 

*Staphylococcus aureus* biofilms on inanimate surfaces are equally problematic, particularly those found in health care facilities and food related industries. Medical devices need to be sterilised and free from all kinds of pathogens. *S. aureus* biofilm on medical devices (made of different materials such as steel, glass, etc.), if not sterilised, can be a major source of infections in hospitals, care homes and GP surgeries [67]. Sterilisation of such surfaces becomes difficult due to *S. aureus*’s resistance against surface cleaners and sanitisers [68]. 

Food industry-related biofilms may encase more than one type of microorganism, including *S. aureus,* and are therefore comparatively more resistant to sanitisers and surface cleaners than the biofilm that encases *S. aureus* cells alone [69]. This microbial resistance to surface cleaners and sanitisers results in food spoilage, contamination of freshly arrived food items and corrosion of storage surfaces, and more importantly, it increases the risk of transmission of infectious diseases [70]. 

*Staphylococcus aureus* biofilm-related infections cause serious life-threatening illnesses in human and animal populations and have incredibly challenging clinical implications that medical scientists have to deal with. 

## 5. Clinical Implications of *Staphylococcus aureus* Biofilms

*Staphylococcus aureus*-related infections are problematic and are difficult to treat due to biofilm formation. The ineffectiveness of the host immune system and antibiotics against *Staphylococcus aureus* biofilm results in the development of chronic infectious diseases [8]. *S. aureus* can form biofilms on different surfaces and medical devices such as surgical instruments, implants, etc., in healthcare facilities. Such surface-related and medical device-associated *S. aureus* biofilms, if not properly sanitised and left untreated, can cause infections in patients [27]. Medical device-associated biofilms, those on implantable devices in particular, often result in the removal and replacement of these devices, thus posing surgical risks as well [8].

*Staphylococcus aureus* also develops biofilms inside the infected host tissues. Such infections are more problematic due to antimicrobial resistance, and their treatment often involves the removal of infected tissues [71]. This biofilm-related antimicrobial resistance is partly due to the presence of some dormant *Staphylococcus aureus* cells (also known as persister cells) encased by its biofilm. These cells maintain their dormancy during antimicrobial treatment and become active as soon as the treatment is withdrawn, thus causing a chronic recurrent infection [72]. 

Treatment of all such aforementioned *S.* *aureus* biofilm-related infections will require novel therapeutic strategies. The first and most important step in this regard is to study *S. aureus* biofilm and understand it thoroughly by employing suitable microbiological and physico-chemical techniques.

## 6. Techniques and Strategies Used in Studying *Staphylococcus aureus* Biofilm

Microbiologists adopt different approaches based on different techniques to study and evaluate *Staphylococcus aureus* biofilm. These techniques are either direct evaluation of biofilm, based on the measurement of thickness of a biofilm, or indirect techniques of measurement where a biofilm is quantified in terms of studying its different constituents or the activity of the bacterial cells within the biofilm [73]. These techniques are discussed in the following sections.

### 6.1. Direct Observation Techniques

Direct observation of the intricacy and changing aspects of biofilms can be studied by biofilm optical imaging technology, comprising light microscopy, scanning electron microscopy (SEM), transmission electron microscopy (TEM) and confocal laser scanning microscopy (CLSM). These methods are employed to examine the presence of biofilm and visualize its 3D structure [74].

#### 6.1.1. Light Microscope and Transmission Electron Microscope

*S. aureus* adhered on cover slips, polystyrene Petri dishes and polymethacrylate films (acrylic sheets) is studied using a light microscope. Observation through a light microscope is incapable of creating a 3D structure but requires a planar and transparent surface for attachment of the microorganism. To improve the image clarity, fluorescent and epifluorescent dyes are used [75].

Cell components can be directly monitored with negative staining using TEM. Glycocalyx of *Staphylococcal* cells are stabilized using cationic reagents such as a combination of alcian blue, ruthenium red, lysine or paraformaldehyde. Following these steps, the sample is ready to be observed under TEM [76]. 

#### 6.1.2. Profilometry and Scanning Electron Microscope

Sterile glass slides were incubated in medium seeded with the test culture. The slides were fixed using Bouin fixative and stained with Alcian blue. Under 100x oil immersion, pictures were taken by photomicroscope with red filter for blue photomicrographs. In order to study the effect of etchant on the biofilm formation and surface morphology of the substrate, this technique can be further modified [77].

The glass slides can be incised/etched chemically to change the surface roughness. The slides are treated with the required etchant at different time intervals followed by incubating the treated slides in overnight test culture. Optical microscope and profilometry were used to measure large fluctuations in thickness of bacterial biofilm and roughness of the substrate surface [78].

To examine the biofilm surface topography and morphology, the test culture was grown in biofilm-promoting agar plates. The mature colonies were removed and the footprints on the agar plates can be visualized under the profilometer [78].

Furthermore, the effect of increasing etching time on biofilm architecture, can be studied by scanning electron microscopy. Therefore, a distinctive correlation between the surface roughness and adherence of bacteria can be studied through this experiment [78].

As SEM is a high vacuum method, before imaging the samples, the biofilm plate was therefore dehydrated. The samples were mounted on SEM specimen stubs and splutter coated with heavy metals, and the electrons which were released from the metal coating were captured by SEM for imaging. The biofilm surface was navigated under the lens and scanned for the desired images [79]. 

This method is also efficient in studying bacterial adherence to submerged solid surfaces, which leads to biofilm formation and hence can be used as a simple model to explore *S. aureus* biofilm.

#### 6.1.3. Scanning Transmission X-ray Microscopy (STXM)

STXM techniques help to explore the nature and extent of macromolecules and dense microbial communities in a biofilm. As a contrast mechanism, STXM uses near-edge X-ray adsorption spectroscopy (NEXAFS) as a new tool for hydrated biological samples [80]. This can be achieved due to the capacity of the X-ray to penetrate water, with decreased radiation damage as compared to electron beam approaches and the presence of appropriate analytic core edges in the soft X-ray sections [81,82]. Soft X-rays offer detection on a scale of 10 nm or less and greater penetration depth, which is appropriate for microorganisms and microbial biofilm imaging [76].

STXM can be coupled with transmission electron microscopy to examine biofilms for correlative mapping and better analysis of spatial distributions of biological macromolecules present in the biofilms [80]. These correlative approaches help in identifying the biochemical sources for the biofilm structure. The analytical ability of STXM answers questions concerning the distribution of carbohydrates, lipids and nucleic acids and the role of proteins in the extracellular matrix of the biofilm [76].

#### 6.1.4. Fluorescent Tagging of Biofilm

Fluorescent tagging of biofilm includes the following:

(a) Confocal laser scanning microscopy (CLSM)

Confocal microscopy has an advantage over SEM in that no dehydration of the sample is required, and the biofilm can be imaged in the hydrated state. CLSM is a great aid to study the physiology and 3D morphology of biofilm [79]. To observe the sample with a confocal microscope, the biofilm can be stained by fluorescence-labelled dyes or tagged with green fluorescent proteins. Biofilm-producing isolate can be engineered genetically by the tagging of microbial gene of interest by gene-cassette-encoding GFP as a reporter gene. These lectin stains are known to target the extracellular matrix, whereas some fluorophores go for eDNA (extracellular DNA), thus assisting us in visualizing the biofilm matrix content [83].

In order to study individual biofilm components, the stains used include SyproOrange for protein, Syto9 for nucleic acids, molecular probes of Nile red for lipid hydrophobic sites and specific lectins for sugar, glycoconjugate and carbohydrate [76,84]. Additional resolution is needed for multi-layered and 3D complex biofilm structures; imaging software is used to combine the layers of biofilms into a digital image.

(b) Fluorescent in situ hybridization (FISH)

Probes of the fluorescent in situ hybridization (FISH) method can help in identifying the *S. aureus* biofilm present in a heterogeneous biofilm community. Fluorescent-labelled cells within biofilm can also be analysed by FISH. DNA probes aimed at hybridizing 16S rRNA of bacteria are incorporated with either an enzyme such as horseradish peroxidase or a fluorescent dye such as FITC or rhodamine [85]. 

An advantage of using probes conjugated with enzyme is that it does not destroy the microbe, and the growth activity within the biofilm can be determined with the number of ribosomes present in a microbe. Probes are intended to label the conserved region of only a specific species. FISH has been applied to explore the 3D distributions of different bacterial activities and examine the thickness of biofilms [86]. 

### 6.2. Indirect Observational Techniques

These include the following:

#### 6.2.1. Tube Method

This is a qualitative technique described by Christensen et al. for the detection of biofilms. A solution of 1% crystal violet was used to stain the biofilm and observe the film around the wall and bottom of the test tube to confirm a biofilm producer. The extent of biofilm formed was recorded as 1: weak/none, 2: moderate and 3: high/strong. Ring formation at the liquid–air interface was not considered indicative of slime production [87].

This technique is frequently subjected to analytical restrictions and is incompetent for detecting bacterial adherence precisely. Therefore, to overcome these shortcomings, the following method can be adapted to modern approaches to obtain precise findings.

#### 6.2.2. Congo Red Agar Method

The Congo red agar screening technique is used to detect whether the *Staphylococcal* isolates are biofilm producers. A positive result is shown by black colonies with a dry crystalline consistency. Weak slime producers are indicated as pink; however, darkening is seen at the centres of colonies occasionally. The blackening of the colonies devoid of dry crystalline morphology suggested moderate biofilm procedures [88].

#### 6.2.3. Detection of Biofilm Production by Microtiter Plate Assay

The microtiter plate assay is a standard quantitative technique to detect biofilm production. The duration of incubation can be modified as needed, and the medium of choice for evaluating the biofilm production is TSB or MHB with 1% glucose. The technique was adapted for better quantification of the biofilms. Optical density (OD) was determined with a microplate reader at a wavelength of 570 nm (OD _570 nm_) [89,90].

#### 6.2.4. Biofilm-Associated Genes Detection by PCR

Biofilm-associated genes are amplified by PCR, such as multiplex, qualitative real-time PCR and conventional PCR, which can identify whether biofilm-associated genes are present in the sample. Instead of qualitative real-time PCR, multiplex and conventional PCR are applied for biofilm gene detection [91]. The isolated PCR product can be visualized on an agarose gel to confirm the amplified gene due to the presence of a DNA intercalating dye, ethidium bromide in the gel, whereas in qualitative real-time PCR, a pair of specific hybridization probes labelled with fluorescence dye is used for detection of the amplicon [92].

#### 6.2.5. Mass Spectrometry

Since the extracellular polymeric substance consists of polysaccharides and proteins, such as extracellular enzymes, these proteins expressed within the EPS matrix are detected and categorized by mass spectrometry [93]. Chemicals and complex biologic molecules involved in the process of biofilm formation can be analysed in detail by MS. Matrix-assisted laser desorption ionization (MALDI) and electrospray ionization (ESI) are the types of MS. By application of MALDI, the expression of proteins and enzymes in response to external factors and the bacterial growth can be monitored. Mass spectrometry is highly sensitivity and can be performed with a minimum amount of sample [75,94]. 

#### 6.2.6. Atomic Force Microscopy (AFM)

Atomic force microscopy is a key technique for imaging samples at the subcellular level. AFM assists in investigating molecular interactions, detecting physiochemical properties such as surface hydrophobicity and quantifying macromolecules on the surface and within the cells [95,96]. The simple principle of AFM is to scan the surface of the sample with a sharp probe tip in a raster pattern; the interaction between the probe tip and the sample is measured on the flexible cantilever. The cantilever bends upon sensing an attractive force on the surface, and this force is measured by gauging the deflection of the cantilever using a photodiode and a laser beam [97,98].

AFM can quantify the adhesion force between the cells, surface and single molecules and is thus used to visualize biofilms. This tool has been applied in gaining an understanding of single and multispecies biofilm components and the core adhesion mechanisms. AFM images allow quantification of biofilm thickness, biomass roughness and EPS content [77]. A detailed insight into the viscoelasticity and adhesion of cells during biofilm formation can help with biofilm control strategies. The viscoelasticity of biofilm influences the penetration of antimicrobials and biofilm removal from the surface and thus plays an important role in protection against chemical and mechanical challenges. This approach can be used to combat antimicrobial resistance in biofilms [98,99]. The aforementioned techniques and their applications have been summarised and listed in Table 2. 

## 7. Strategies Used to Inhibit and Disrupt *Staphylococcus aureus* Biofilm

Biofilms are a major cause of antimicrobial resistance and complications in treating *S. aureus*-related infections [111]. It is, therefore, important to treat these biofilms to minimise and curtail the chances of antimicrobial resistance by adopting adequate antibiofilm strategies. Antibiofilm strategies are mainly of two types, i.e., the inhibition or prevention of new biofilm formation and the dispersal or eradication of existing biofilms [112].

Different antibiofilm agents including drugs, functional excipients and other antibiofilm molecules (both naturally occurring and synthetic) have been reported to serve the purpose of biofilm inhibition and its disruption in *Staphylococcus aureus*. These antibiofilm agents are discussed in the following sections. 

### 7.1. Antibiofilm Drugs and Functional Excipients

Several publications have been reported to have provided information about antibacterial drugs with antibiofilm activities. Rifampin, for instance, an antibiotic used for the treatment of TB and other bacterial infections, has been reported to have inhibited and disrupted biofilm in *S. aureus* [113,114]. Rifampin has also been reported to have shown antibiofilm activity in combination with another antibiotic, ciprofloxacin, against *S. aureus* biofilm [115]. 

Similarly, vancomycin, an antibacterial drug used in the treatment of both methicillin-resistant *Staphylococcus aureus* (MRSA) and methicillin-susceptible *Staphylococcus aureus* (MSSA), has been reported to effectively treat *Staphylococcus aureus* biofilms [112,116]. Vancomycin has been used in combination with other drugs to treat *Staphylococcus aureus* biofilm, e.g., a combination of vancomycin and rifampin as antibiofilm dual therapy in MRSA-related infections [117]. Hu et al. [118] reported the use of the antibiotics azithromycin and clindamycin for the inhibition and dispersal *S. aureus* biofilm. The antibiofilm mechanism of azithromycin is based on its ability to disrupt bacterial quorum sensing [119].

The mechanisms by which antibiotics inhibit and or disrupt *Staphylococcus aureus* biofilm are not fully known and have not been reported thus far. Therefore, the antibiofilm mechanisms of antibiotics still remain an area that needs to be explored in order to devise effective strategies for treating *S. aureus* biofilm-related infections.

Apart from antibiotics, other naturally existing compounds such as amino acids, antimicrobial proteins (AMP), polyether ionophores, essential oils and plant extracts have also been used to inhibit and eradicate *Staphylococcus aureus* biofilm.

Scientists have used amino acids as antibiofilm agents either (1) as individual amino acids, (2) as mixtures of different amino acids or (3) as combinations of amino acids and antibiotics. Sanchez et al. [120], for instance, used D-amino acids as individual amino acids, equimolar amounts of D-amino acids as a mixture and also a D-amino acids-antibiotic combination to inhibit and disperse *Staphylococcus aureus* biofilm. One similar study conducted by Warraich et al. [121] employed a combination of amino acids and the antibiotic ciprofloxacin, which not only successfully inhibited the formation of new biofilm but also disrupted the existing biofilm in *Staphylococcus aureus*. 

The antibiofilm activity of D-amino acids involves their ability to inhibit the formation of biofilm-related proteins and disruption of eDNA. Hochbaum et al. [122] experimentally assessed the role of D-amino acids in inhibiting the formation of biofilm-related proteins in *S. aureus*. Similarly, Warraich et al. [121] used confocal microscopy and showed that the antibiofilm mechanism of D-amino acids involved their ability to disrupt eDNA in the EPS matrix of *S. aureus* biofilm.

Amino acids, when linked together, form protein polymers. Different proteins have been reported with antimicrobial properties. These antimicrobial proteins (AMP) have also been the target of scientific exploration for their antibiofilm activity, either alone or in combination with other antibiotics. For example, Nair et al. [123] have used a bactericidal protein P128 in synergy with different antibiotics, such as vancomycin, gentamicin, linezolid and daptomycin, to inhibit the formation of *S. aureus* biofilm. 

The antibiofilm mechanism of AMPs involves (i) the prevention of gene expression encoding the formation of biofilm-related proteins and (ii) AMPs’ attachment to eDNA [17].

Scientists have also been exploring the antibiofilm activity of naturally occurring polyether ionophores. One such study carried out by Hickey et al. [124] used four different polyether ionophores, lasalocid, monensin, narasin and salinomycin, and successfully eradicated *Staphylococcus aureus* biofilm. Although, the antibiofilm activity of polyether ionophores has been reported, the exact mechanism of action needs to be investigated.

Alongside the aforementioned antibiofilm approaches, recent years have seen scientific work focussing on the use of essential oils and plant extracts amongst the future’s effective antibiofilm therapies. Bazargani and Rohloff [125] have reported the use of different essential oils and plant extracts to inhibit and disperse *Staphylococcus aureus* biofilm. 

Essential oils and plant extracts have also been used in combination with antibiotics against *S. aureus* biofilm, e.g., Monteiro-Neto et al. [126] studied Cuminaldehyde, a natural compound found in essential oils, in combination with ciprofloxacin and reported its synergetic antibiofilm activity against *S. aureus* biofilm. The antibiofilm mechanism through which plant extracts and essential oils target *S. aureus* biofilm involves the disruption of bacterial quorum sensing [119,127].

### 7.2. Other Antibiofilm Molecules

Antibiofilm molecules are small molecules (other than the drugs and functional excipients discussed in Section 6.1) exhibiting antibiofilm activity. Unlike the functional excipients (most of which are antibacterial as well), these molecules are not antibacterial but are antibiofilm only [128]. These molecules include imidazoles, indoles, pyrazoles, carbazoles, pyrroles, oxazolidinones and furanones, derivatives of phenazine, cinnamide and quinoline, etc. [129]. The molecular mechanism of antibiofilm activity of these small molecules is not yet fully known.

Apart from these small molecules, enzymes, such as DNA and exopolysaccharide degrading enzymes, and nanoparticles of heavy metals, such as silver and zinc, have also been used to prevent *Staphylococcus aureus* biofilm formation. The enzymes DNase I and Dispersin B have been reported to have degraded eDNA, while the enzyme a-amylase has been reported for its degradation of exopolysaccharides in *S. aureus* biofilm [119].

Nanoparticles have also been used either in isolation or in combination with other antibacterial drugs for the inhibition and dispersal of *S. aureus* biofilms. For instance, Fontecha-Umaña et al. [130] used silver and zinc oxide nanoparticles on their own, while Hamida et al. [131] reported a combination of silver nanoparticles and antibiotic ampicillin as an antibiofilm strategy for *Staphylococcus aureus* biofilm. The antibiofilm activity of nanoparticles has been reported in numerous studies; however, their mechanism of action has yet to be explored. 

The antibiofilm agents mentioned in Section 6.1 and Section 6.2 have different mechanisms of actions for the inhibition and/or dispersal of *Staphylococcus aureus* biofilm. These mechanisms include the obstruction of bacterial adhesion and quorum sensing; disruption of extracellular DNA (eDNA), proteins, lipopolysaccharides, exopolysaccharides; and inhibition of signalling molecules, i.e., N-acyl homo-serine lactones [74,129]. Table 3 summarises different kinds of antibiofilm agents and their mechanisms of action.

## 8. Conclusions and Future Perspectives

Microorganisms including *S. aureus* have the remarkable ability to enclose themselves in a protective environment, i.e., biofilms, to survive and cause recalcitrant infections. The primary focus of this review was to discuss the different genetic and morphological factors that influence biofilm formation in *S. aureus* specifically and their clinical implications. As highlighted in this review, techniques such as microtiter plate assay coupled with crystal violet staining, CLSM and mass spectroscopy provide high throughput detailed information on biofilm morphology in the normal hydrated state and aid in the characterisation of individual EPS components, respectively.

Despite the advances made in studying the composition, architecture and dynamics of biofilm formation in *S. aureus,* little is known about the mechanism of action of antibiofilm agents used to inhibit or disrupt these biofilms. There is also a dearth of information on the effect of antibiofilm agents and their mode of action on the physiology of cells residing in different layers of the biofilm. Further research is needed to fill in these knowledge gaps. The most interesting question to answer would be whether these antibiofilm agents have specific targets (EPS proteins/polysaccharides, eDNA, persister cells, etc.) or whether they have multiple mechanisms for biofilm inhibition and dispersal. Deciphering the precise mechanism will predict if nosocomial pathogens, such as *S. aureus*, will develop resistance to these novel agents or not. 

Novel antibiofilm and antimicrobial combinations have the potential to hinder the development of resistance in microorganisms if they have multiple modes of action. Therefore, elucidating the multimodal effects of functional excipients, such as amino acids and antibiofilm agents, will inform the design and development of these drug combinations. This will lead to the targeted use of these agents to treat a wide range of biofilm-related infections caused by this notorious pathogen and eventually strengthen the fight against AMR.

## Figures and Tables

**Table 1:** A list of different virulence factors, their corresponding regulatory genes and their resultant clinical implications. Data have been collected from Otto (2014), Gordon (2008) and Oogai (2011) [47,48,49].

Virulence Factors		Genes	Clinical Implications
Toxins	Hemolysin	*Hla*	Food poisoning, toxic shock syndrome, scalded skin syndrome, bullous impetigo and sepsis syndrome
	Leukotoxin	*lukD* and *E*	
	Exfoliative toxin	*eta* and *etb*	
	Toxic shock syndrome toxin 1	*tstH*	
Exoenzymes	Lipases	*Geh*	Tissue destruction and metastatic infections
	Proteases	*aur, sspA, B* and *C*	
	Nucleases	*nuc1* and *nuc2*	
	Coagulases	*coa* and *vWbp*	
	Hyaluronate lyase	*hysA*	
Immunomodulators	Leucocidin	*lukS-PV* and *lukF-PV*	Invasive skin infections, pneumonia and abscesses
	Extra cellular adherence protein	*Eap*	
	Capsular polysaccharides	*cap5* and *cap8*	
	Phenol-soluble modulins	*psm-* *α*	
Other	Attachment	*clfA* and *B, fnbA* and *B, cna* and *ica*	Endocarditis, septic arthritis, prosthetic devices and catheter infections, cystic fibrosis and relapsing infections
	Persistence	*Ica locus* and *hemB*	

**Table 2:** Summary of techniques used for biofilm visualization and characterisation.

Sr. no	Techniques	Summary	Advantages	Disadvantages	References
a.	Direct observation techniques				
1.	Light microscope	Visualization of biofilm structure.	Cheap, convenient and easy to perform. Simple sample preparation.	Restricted resolution and magnification. Sample staining required. Lacks discriminatory features.	[97,100]
2	Transmission electron microscope	Images of cell components on the biofilm surface and within the matrix are directly visualized with negative staining.	Biofilm labelling. Several observational modes such as nanometric scale and elemental evaluation.	Expensive method. Long fixative procedure. Possible detachment of biofilm during fixative procedures.	[101]
3.	Scanning electron microscope	Two-dimensional and topographical imaging of biofilm structure.	Allows to examine the biofilm matrix on the growth substratum. Presents observational modes such as nanometric scale imaging. High magnification and resolution.	Expensive and high maintenance. Risk of artifacts. Samples must be solid and non-conductive. Conductive material coating and dehydration causes biofilm shrinkage. Time-consuming technique.	[97,99,102]
4.	Scanning transmission X-ray microscope	Quantitative and qualitative explorations of biofilm structure and analysing the array of microbial communities.	High resolution. Quantitative mapping of biofilm components such as lipids, proteins, nucleic acids and saccharides. Provides spectral outline for each component.	Limited accuracy. Risk of instrumental systematic error. Utilized on thin samples.	[76,103]
b	Fluorescent tagging of biofilm				
1.	Confocal laser scanning microscope	Three-dimensional morphology and quantitative imaging of biofilm physiology.	Non-invasive technique. Living, hydrated samples.	Interference of biofilm components with the fluorescence probes. Restricted number of reporter molecules. Limited choice of magnification.	[76,97,104]
2.	Fluorescent in situ hybridization (FISH)	Semi-quantitative technique to identify specific organism in a multispecies biofilm population with fluorescent probes.	Applicable to heterogenous biofilm community. Detection of live microorganisms.	Low sensitivity due to non-specific hybridization of complementary probes. Tedious procedure and expensive requirements.	[103,104]
C	Indirect observational technique				
1.	Tube method	Qualitative detection by presence of visible biofilm lining around the wall and the bottom of the tube.	Identifies strong biofilm producers.	Fails to differentiate between weak, moderate or non-biofilm producers due to the variability in the findings identified by different viewers.	[105]
2.	Congo Red agar method (CRA)	Qualitative method by examining the colony colour change on Congo red agar (CRA) medium.	Cheap and easy to perform.	Substantial low specificity, sensitivity and positive predictive value.	[105,106]
3.	Microtiter plate assays	Quantitative evaluation of biofilm formation in the wells detected by microplate reader.	Quick and simple screening assay to quantify the biofilm formation. Antimicrobial susceptibility assay	Low reproducibility. Non-specificity with crystal violet dyes. Variation in biofilm biomass, depending on the washing step. Limited substratum alternatives.	[107,108]
4.	Biofilm-associated genes detection by PCR	Detection of biofilm-associated genes in microorganisms	Presents sharper specificity, sensitivity and time efficient. Reliable and reproducible	Possibility of sample contamination, false positive results or misinterpretation. High-priced PCR requirements.	[103,109]
5.	Mass spectrometry	Detection of proteins and enzymes expressed with EPS matrix.	Provides identification of proteins, chemical components and mass-based variation of analogous molecules. Detection of chemical heterogeneity and secondary metabolites, even in multispecies biofilms. Cell-level and macroscopic chemical alterations.	Imaging artifacts. Sample surface requires chemical modification.	[103,110]
6.	Atomic force microscopy	Utilized to map distributions of EPS, biomass, chemical and molecular compounds with a physical probe tip scanning the sample surface.	Minimal pre-treatment procedures and artifacts. Three dimensional images. Qualitative imaging of EPS. High resolution. Elucidation of cellular and molecular interactions. Imaging samples at the nanometre to micrometre scale.	Small scanning area (max 150 × 150 µm). Risk of surface damage due to sample-probe tip interactions.	[77,95,96,97]

**Table 3:** Different categories of antibiofilm agents with examples and reported mechanism of action against *S. aureus* biofilm.

Antimicrobial Agents	Examples	Mechanism of Action Against *S. aureus* Biofilm
AMPs	NA-CATH: ATRA1-ATRA1	Prevention of gene expression that encodes the formation of biofilm-related proteins. Attachment to eDNA
Functional Excipients	D-amino acids	Disruption of eDNA
Plant Extracts and Essential Oils	Garlic and ginseng extracts	Disruption of bacterial quorum sensing
	*Melaleuca bracteate* leaves oil	Inhibition of biofilm-related proteins formation and disruption of quorum sensing
Enzymes	DNase I, Dispersin B	Degradation of eDNA
	a-amylase	Degradation of exopolysaccharides
Nanoparticles	Silver, zinc oxide	Unknown
Antibiotics	Azithromycin	Inhibition of EPS related proteins formation and disruption of quorum sensing
	Ciprofloxacin, rifampin, amoxicillin, clindamycin, vancomycin, etc.	Except azithromycin, mechanism of action for antibiofilm activity of most of these antibiotics is not fully understood
Ionophores		Unknown
